# Urgent Removal of a Mobile Mass in the Ascending Aorta under Hypothermic Circulatory Arrest in a Patient with Acute Stroke: A Case Report

**DOI:** 10.1055/a-2536-4259

**Published:** 2025-05-08

**Authors:** Jenna E. Aziz, Jesica Zvara, Cathy Burger, Shawn Sarin, Salim Aziz

**Affiliations:** 1 Department of Surgery, Ohio State University Medical Center, Columbus, Ohio; 2 Department of Anesthesia, George Washington University, Washington, District of Columbia; 3 Division of Neurology, George Washington University, Washington, District of Columbia; 4 Department of Radiology, George Washington University, Washington, District of Columbia; 5 Divison of Cardiac Surgery, George Washington University, Washington, District of Columbia

**Keywords:** aortic surgery, ascending aorta, hypothermic circulatory arrest, acute stroke, cardiopulmonary bypass, computed tomography angiography

## Abstract

A mobile mass in the ascending aorta is a rare cause for stroke. Detection is usually accomplished by Computed tomography angiography and/or echocardiography. In suitable patients, urgent surgical removal remains the best approach.

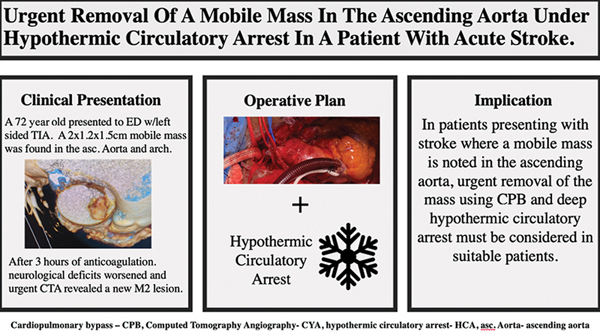

## Introduction

The ascending aorta and the aortic arch are very infrequent sources of embolic strokes. Early identification of these sites as a source is essential in order to select optimal therapy and prevent recurrence. 1 2 It is advisable to include the ascending aorta and the arch in computed tomography angiography being done for stroke evaluation. The information gathered can assist with innovative approaches in stroke management, even in the elderly.

Once detected, the management of a mobile mass in the ascending aorta and arch presenting with a stroke is a medical emergency. 2 Evidence regarding the optimal time interval between stroke and cardiac surgery is conflicting. The management of such lesions is not well defined and requires a multidisciplinary approach. There is a danger of conversion of a bland to a hemorrhagic stroke on cardiopulmonary bypass (CPB). 3

In suitable cases, surgical removal is expedient, requires CPB, and possible deep hypothermic circulatory arrest (HCA) and replacement of various parts of the aorta. 4 Furthermore, the effects of addition of deep HCA with brain perfusion techniques to protect the brain in the presence of a recent stroke has not been well reported. We report our urgent successful approach using CPB and moderate HCA in managing such a case.

## Clinical Presentation

**Video 1** Computed tomography angiography shows a mass in the ascending aorta adjacent to the pulmonary artery.


**Video 2** Repeat computed tomography angiography performed 3 hours after occurrence of new right-sided hemiplegia shows occlusion of left M2.


**Video 3** Intraoperative transesophageal echocardiogram shows mobile mass in ascending aorta.


**Video 4** Postoperative computed tomography angiography of ascending aorta. No mass present.


**Fig. 1:**
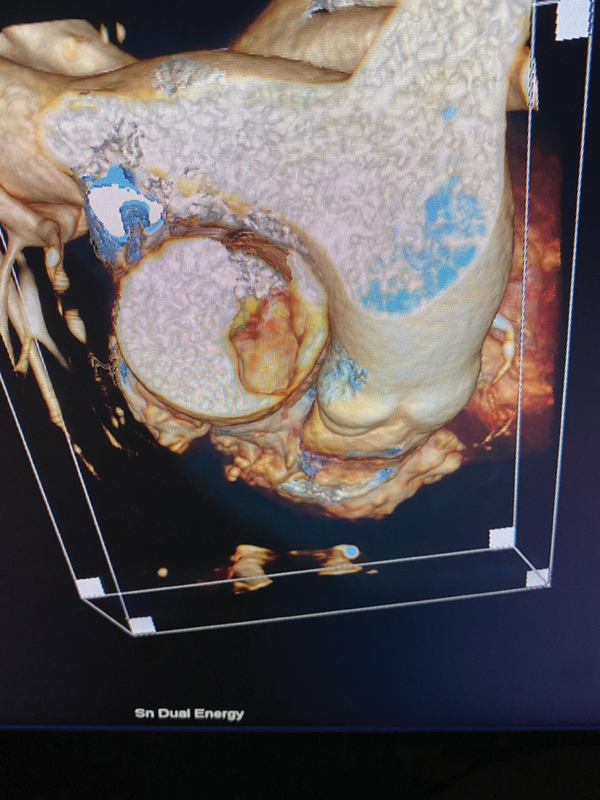
Computed tomography angiography mass in ascending aorta.

A 72-year-old African American female with a long history of heavy smoking presented to the emergency room with left-sided hemiparesis and left lower leg cramps. As part of her stroke workup, a computed tomography and a CTA were done. Fortuitously, scanning included the proximal aspect of the ascending aorta. This revealed a mass ( Fig. 1 and Video 1 [available in the online version]). There was no intracranial acute pathology detected and no evidence of a hemorrhage. There was no evidence of carotid stenosis or ulceration. The patient was in sinus rhythm. She was started on intravenous heparin after a multidisciplinary discussion, which included cardiovascular surgery. While the patient was being prepared to be moved to the stroke unit, her left-sided hemiparesis improved, and she developed a new right-sided hemiparesis. A repeat CTA of the head/neck and ascending aorta was performed. Now, there was a left distal M2 cutoff ( Video 2 , available in the online version) and the mass in the ascending aorta was smaller.

These findings suggested that the ascending aortic lesion was embolizing to the brain despite heparin therapy. At a multidisciplinary meeting, interventional neuroradiology opined that the embolus in M2 could not be aspirated. Cardiac surgery agreed to surgically remove the embolic source in the ascending aorta, accepting the risk of hemorrhagic conversion of bland infarct.

In the operating room, transesophageal echocardiogram (TEE) ( Video 3 , available in the online version) confirmed the aortic mass. Inspection after median sternotomy revealed severe calcification in the distal ascending aorta and the proximal arch. This precluded cannulation in those areas as well as the use of an aortic cross-clamp.

**Fig. 2:**
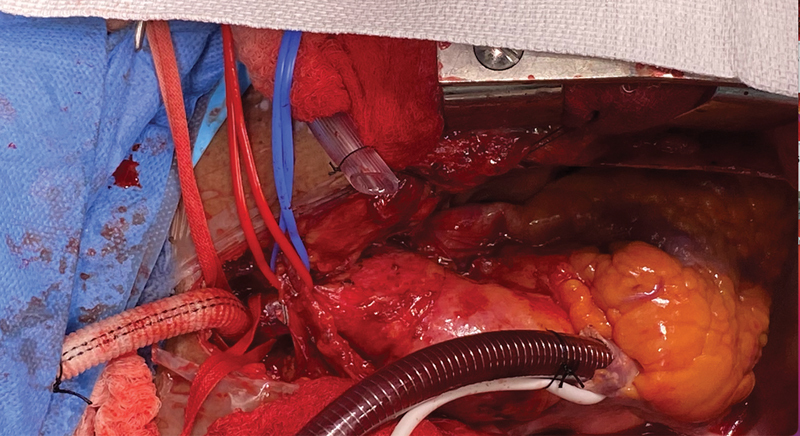
An 8-mm hemashield graft anastomosed to the Innominate artery to allow provide arterial inflow site for cardiopulmonary bypasses as calcified ascending aorta and proximal arch.

We elected to use the innominate artery as the site for cannulation for CPB. A graft was anastomosed to the innominate artery with neurological monitoring in progress ( Fig. 2 ) Because of the inability to cross-clamp the aorta, we used hypothermia circulatory arrest. At 22°C, the circulation was stopped and the blood drained into the pump. Brain protection was accomplished under HCA by means of antegrade cerebral perfusion, steroids, and ice-packs to the head.

**Fig. 3:**
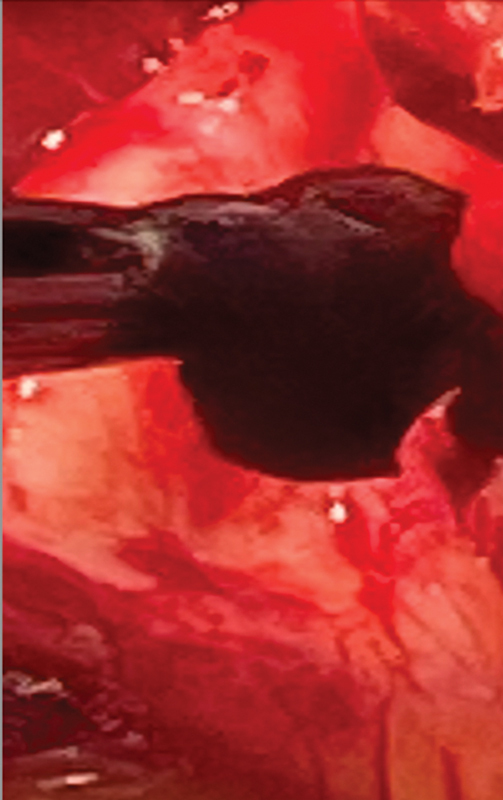
Gelatinous black mass in ascending attached to wall of ascending aorta. Histology after removal was a thrombus.

The ascending aorta was incised in a soft location, and, with heavy scissors, the calcified portion of the aorta was cut open. The gelatinous black mass was removed from the lower portion of the ascending aorta ( Fig. 3 ). The aortic valve did not have any vegetations. The left ventricle and left atrium were thoroughly irrigated with cold saline and no mass/debris was identified. The TEE did not show any thrombus in the auricular appendage.

**Fig. 4:**
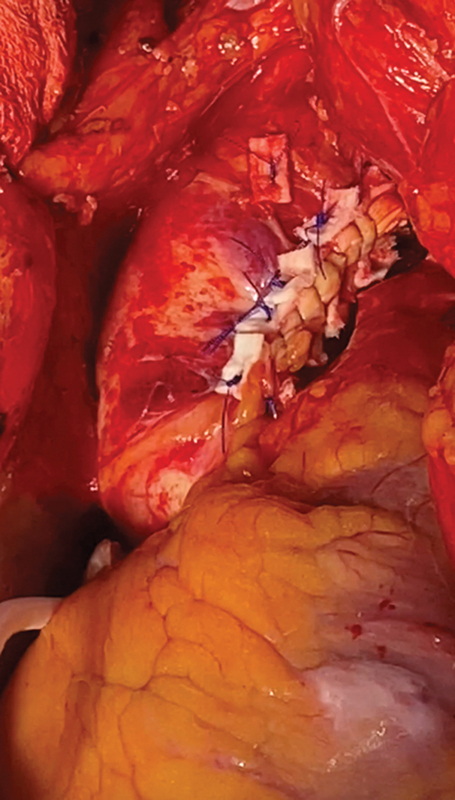
After ascending aorta and proximal arch endarterectomy, the aorta was closed.

During the period of HCA, no thrombus was noted in the arch. However, there were multiple areas of ulcerated calcified plaque with protrusions in the distal ascending aorta and inner curve of proximal arch. An endarterectomy of these areas was done to allow closure of the area of incision and prevent potential embolization ( Fig. 4 ). The patient was weaned off CPB uneventfully and made an excellent and complete neurological recovery. Histology of the mass showed a thrombus.

Postoperative CTA ( Video 4 , available in the online version) showed complete removal of the ascending aorta mass. The patient did complain of ongoing left lower leg and foot pain and required transfemoral embolectomy.

## Discussion

The pathology of stroke is broadly divided into hemorrhagic (10%) and ischemic (90%). 5 The initiating pathology may be intracranial or embolic from a cardiac source. The ascending aorta and the arch and its branches are very infrequent sources for stroke. 2 Hence, in the initial imaging studies, these zones are not routinely included.

Virchow's triad outlines the interplay of factors contribution to thrombus formation. The de novo occurrence of thrombus in the ascending aorta, in the absence of wall pathology, is highly unusual due to the high-flow dynamics in this location. At the time of presentation, our patient had no antecedent history of clot formation.

Our case suggests that one must remember to consider the ascending aorta and aortic arch as possible causes of ischemic stroke, especially in the elderly and patients with underlying thrombotic tendencies. Aortic CTA plays an important role in the diagnosis and treatment of such aortic thrombi. Another advantage of such imaging is that this will also help detect acute Type A dissections as a potential cause of the stroke.

A number of possible approaches have been used to manage a thrombus in the aorta. These include sole anticoagulation, lytic therapy, aspiration thrombectomy, and direct thrombectomy. In a patient who has ongoing evidence of repeat embolization from a mobile floating thrombus in the ascending aorta, options are limited. 6 7 All the aforementioned techniques can result in further embolization to the brain. In this situation, and in a patient who can tolerate cardiopulmonary bypass and possible circulatory arrest, urgent direct surgical removal is essential.

Evidence regarding the optimal time interval between stroke and cardiac surgery is conflicting. During the acute phase of an ischemic stroke, the need for full anticoagulation during cardiopulmonary bypass and hypotension may result in risk of hemorrhagic transformation due to breakdown in the blood–brain barrier and extension of brain injury. 8 9 The timing to safely place a patient on CPB after an ischemic stroke has been reduced from the early recommendation of 6 weeks to, in some cases, hours. 10 11

Particular attention must be paid to arterial cannulation strategies. It would be dangerous to place the arterial perfusion cannula directly in the aorta if the clot is in the ascending aorta or the aortic arch. We favor placing the arterial cannula into the innominate artery, thus allowing antegrade-directed aortic flow. Having a cannula in the innominate artery also allows for antegrade cerebral perfusion during HCA.

Others have used retrograde perfusion via the femoral artery. This has a propensity for causing retrograde coronary and or cerebral embolism of the floating thombus. 12 Kalangos et al have described successful removal of the arch thrombus without HCA, by using of selective cerebral perfusion in combination with retrograde femoral artery perfusion. 7

If a mass has been detected in the proximal ascending aorta, an aortic arch cross-clamp can be applied. Also, retrograde cardioplegia should be used to prevent coronary embolization.

The incidence and variety of neurological injuries associated with deep HCA and reasons for the various patterns of injury and measures to reduce the incidence have been well described. 13 However, we were unable to find any article or opinion in the literature about the safety of using DHCA in patients with a very recent embolic stroke.

The need for concomitant aortic arch and ascending aortic endarterectomy was evident after aortotomy. The other option was performance of a total arch replacement, which, in this frail, elderly lady with a recent stroke, would have been associated with increased morbidity and mortality.

Histology of the removed ascending aortic mass showed an organized thrombus ( Fig. 3 ). and no evidence of infection, inflammation, atherosclerosis, or malignancy. The patient did have a history of COPD, heavy smoking, and hypertension. There was no ulceration seen at the site of the attachment of the thrombus to the ascending aorta. The endartectomy specimens of the arch showed classical atherosclerotic findings.

We believe this case shows that in patients with ischemic embolic stroke who require CPB and have a hostile aorta: (1) moderate HCA and alternate cannulation strategies for CPB should be considered to deal with ascending aortic and arch-related pathologies; (2) HCA does not exacerbate brain injury in a patient even with a very recent embolic stroke; and (3) early intervention can reverse stroke symptoms, with a favorable outcome. The patient continues to do well and is on oral thrombin inhibitor and Plavix.

A thrombus in the ascending aorta is an unusual cause of ischemic strokes. Once detected, in suitable patients, urgent surgical removal must be considered. Our approach suggests that CPB and HCA can be used safely, early, even in the presence of a recent stroke, and a hostile aorta. This approach permits aortotomy, clot removal, and reversal of neurological deficits.
